# Differential diagnosis and pathogenesis of the neurological signs and symptoms in COVID‐19 and long‐COVID syndrome

**DOI:** 10.1111/cns.13957

**Published:** 2022-09-19

**Authors:** Abdul Mannan Baig

**Affiliations:** ^1^ Department of Biological and Biomedical Sciences Aga Khan University Karachi Pakistan

## Abstract

Neurological features have now been reported very frequently in the ongoing COVID‐19 pandemic caused by SARS‐CoV‐2. The neurological deficits associated features are observed in both acute and chronic stages of COVID‐19 and they appear to overlap with wide‐ranging symptoms that can be attributed to being of non‐neural origins, thus obscuring the definitive diagnosis of neuro‐COVID. The pathogenetic factors acting in concert to cause neuronal injury are now emerging, with SARS‐CoV‐2 directly affecting the brain coupled with the neuroinflammatory factors have been implicated in the causation of disabilities in acute COVID‐19 and patients with Long‐COVID syndrome. As the differentiation between a neural origin and other organ‐based causation of a particular neurological feature is of prognostic significance, it implores a course of action to this covert, yet important neurological challenge.

COVID‐19 has challenged clinical experts from diverse disciplines in the management of patients with end‐organ COVID‐19 complication‐related clinical features, which are at times seen to be more disabling than lethal. Central nervous system (CNS) manifestations have been reported now with lesions affecting the brain in particular in the acute phase of COVID‐19^1^ as well as the chronic long‐COVID syndrome.^2^ The patients with residual effects of the COVID‐19, which persist beyond three months, are presenting with complaints in which the neuropsychiatric sequelae were found to be prevalent over others.^2^ Early in the COVID‐19 pandemic, the autopsies of the lungs, heart kidneys, and blood vessels were frequently done but the CNS tissue got overlooked initially for histopathological examination, despite the reports that SARS‐CoV‐2^1^, ^3^ may be targeting the brain and causing neurological deficits. It is important to recognize that not only does the neurological involvement in COVID‐19 remains important to be spotted to prevent the consequent disabilities it can cause,^1^, ^2^ but a timely diagnosis of lesions affecting the brainstem if diagnosed can prevent fatal outcomes in COVID‐19.^4^ Another problem while evaluating CNS‐related features in COVID‐19 and Long‐COVID is the dangers of associating casual syndromic presentation with authentic neurological signs and symptoms that can be precisely attributed to an area in the brain or spinal cord. The latter problem and conceivable approaches to tackle it are highlighted here because a considerable overlap between these features can overdiagnose or underdiagnose the patient.^1^, ^2^ The fact that once multiorgan involvement in COVID‐19 (Figure 1A) is in effect,^3^ several etiological factors can evoke signs and symptoms in concert which can be problematic to segregate. There is a possibility of erroneously allocating the non‐neurological features in COVID‐19 to the CNS and vice‐versa (Figure 1). Concerning this, spotting a peculiar clinical feature to be of neural or non‐neural origin in COVID‐19 appears to be critical but at the same time is challenging. The majority of patients with neurological derangements have been noted to present with central fatigue (up to 87%), intellectual dysfunction (“brain fog”), pain (e.g., headaches, myalgia), and emotional dysregulation (e.g., anxiety, depression),^2^ also it is disturbing to note that the estimated overall probability of a diagnosis of a new psychiatric illness within 90 days after COVID‐19 diagnosis is 5.8% (anxiety disorder = 4.7%; mood disorder = 2%; insomnia = 1.9%; dementia [among those ≥65 years old] = 1.6%) among a subset of 44,759 patients who had no known previous psychiatric illness.^5^

**FIGURE 1:**
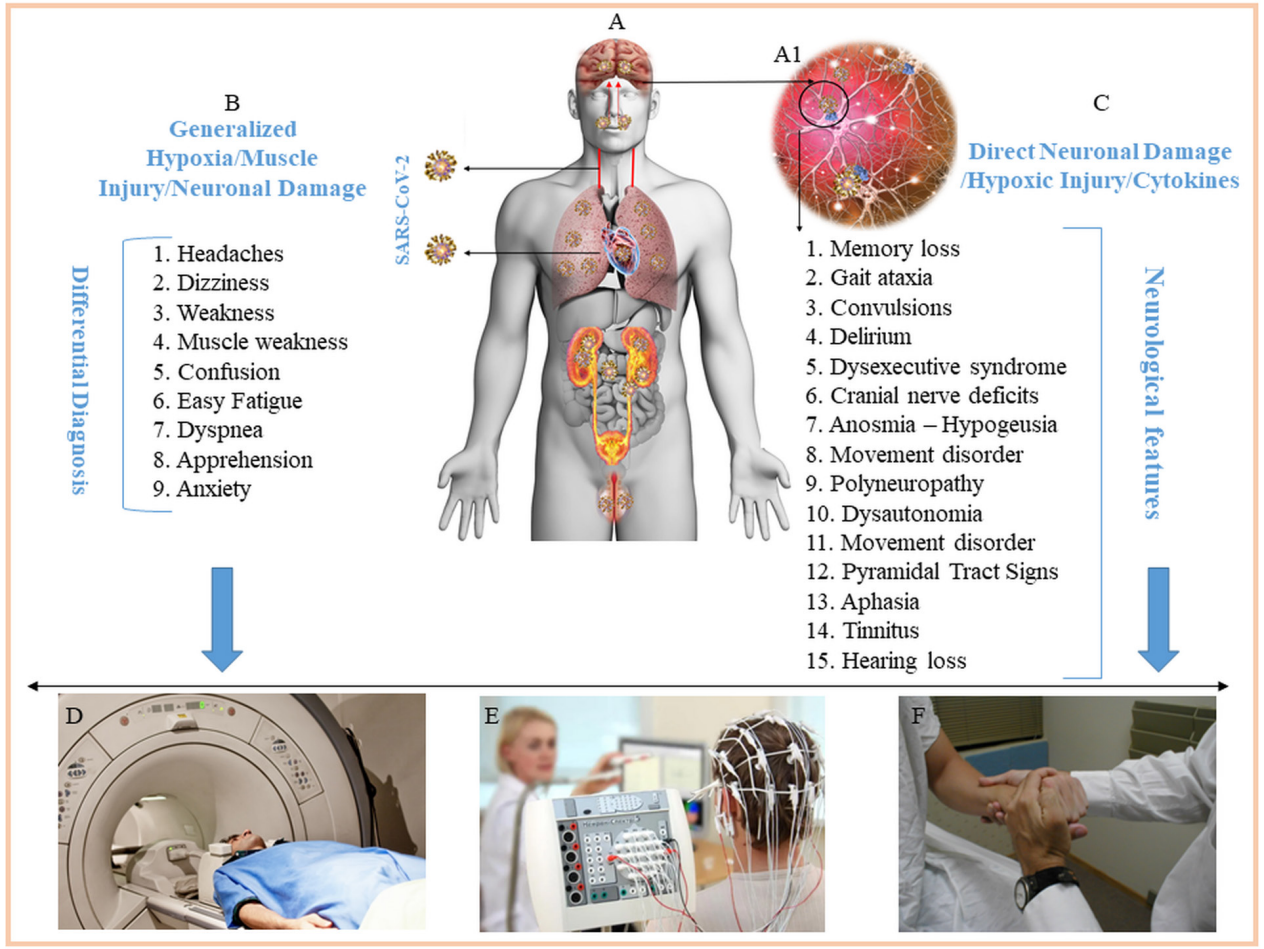
Schematic diagram illustrating the overlapping neurological signs and symptoms of COVID‐19. SARS‐CoV‐2 can use various routes to reach the CNS like the bloodstream and across the cribriform plate (A‐arrows). The neuronal damage (A1) can elicit generalized signs and symptoms (B), which can be due to non‐specific generalized hypoxia, muscle injury, cytokines, or neuronal damage (C). Syndromic features can be due to specific neuronal damage (A‐inverted arrow). Specific neurological involvement can be included or excluded by an investigation like MRI, EEG, and a thorough clinical examination (D–F).

Evaluation of the COVID‐19 patients with neurological complaints using current modalities like electroencephalograms and neuroimaging^1^, ^3^, ^4^ like functional MRI (fMRI) and meticulous methodical neurological clinical examination, a few of many other approaches, is expected to spot patients with specific neurological deficits as has been reported recently.^1^, ^2^, ^3^, ^4^, ^5^ Features like confusion, apprehension, headaches, visual disturbances, dizziness, fatigue, myalgia, and brain fog, all of which have been listed as the neurological features in COVID‐19 in reported studies can occur because of generalized hypoxia that ensues due to the pulmonary exudate or pulmonary fibrosis in COVID‐19. Importantly, a few of the aforesaid features also are a part of the presentation of neuro‐COVID in patients with Long‐COVID.

Blood PO_2_ and PCO_2_ assessment with radiological examination of the lungs for opacities and scarring can serve as a useful guide in the differential diagnosis of symptomatic patients with neurological features, as alteration of these gases may explain the vague presentations (Figure 1B) in COVID‐19. Patients with Long‐COVID have also been reported with neurological complaints like “brain fog,” which is a cognitive deficit, and features of nerve damage possibly involving spinal and cranial nerves are states where a similar differential diagnosis is needed to distinguish neural and non‐neural causes as the basis of their syndromic presentations. In instances where a discrete sign or symptom hints towards a specific CNS area, the diagnosis can be made by neuroimaging techniques coupled with neurological examination, as mentioned above, which can exclude the non‐neural organ and system for the origins of the presenting symptoms. Shown in Figure 1 is a summary of neurological features that can be investigated in the differential diagnoses with lesions either credited to a particular location in the CNS or factors like hypoxia, ischemia, muscular injury, and systemic homeostatic dysregulation.

It was felt contextual and significant to synopsize the root causes that are capable of producing focal neurological lesions which can be the basis of discrete neurological deficits seen in COVID‐19 and Long‐COVID. It has been over a year in the quest to spot the underlying cause(s) that are the basis of neurological features in COVID‐19.^1^, ^6^ It appears that (a) The SARS‐CoV‐2–mediated direct neuronal damage^1^ and indirect causes like (b) hypoxia, breaches in the blood–brain barrier, and inflammatory cytokines^7^, ^8^ appear to be acting in concert to produce the neurological complications seen in COVID‐19 and Long‐COVID. Studies have shown neurochemical evidence of neuronal injury with glial activation in patients with moderate and severe COVID‐19, which has hinted strongly towards neuronal axonal injury with elevated levels of neurofilament light chain protein and glial fibrillary acidic protein.^6^ Several recent reports are emerging that have identified SARS‐CoV‐2 to infect the neurons and brain organoids,^8^ which along with elucidation of adapter proteins like ACE2 and TMPRSS‐2^9^ and NRP‐1 hint towards the neurotropic potential of SARS‐CoV‐2 heralded in the early days of COVID‐19 outbreak in Wuhan, China.^1^, ^10^

In the future, pieces of evidence elucidating neuronal injury caused by SARS‐CoV‐2, with unequivocal evidence of viral budding from neurons undergoing cell death, is expected to further clarify the direct role of SARS‐CoV‐2 in the causation of neurological manifestation of COVID‐19. It remains obscure why many Long‐COVID patients continue to exhibit neurological features,^11^ it appears to be the effects of a residual SARS‐CoV‐2, the effects of a cerebral hypoperfusion state, or both. It is also imperative to mention here the significance of an early diagnosis of symptoms heralding neurological deficits in COVID‐19 and Long‐COVID, as once the imaging modalities like CT and MRI scans begin to exhibit the changes like loss of cerebral or cortical mass, demyelination, and spinal cord damage, the changes could not be reverted.^12^

In conclusion, regardless of the mechanisms underlying the neuronal damage, the finding of neurological complications of COVID‐19 in the acute phase and Long‐COVID syndrome is alarming. A distinction of the focal neuronal zone(s) affected causing a specific neurological problem should be identified whenever possible. The healthcare professional should be mindful of differential diagnoses considering the roots of the origins of the adverse syndromic neurological outcomes in COVID‐19 and Long‐COVID. It is also worth considering that in COVID‐19 and Long‐COVID, a slow smoldering inflammation or apoptosis in neurons set by SARS‐CoV‐2 could be covert symptomatically in the early course of the disease that later becomes evident in the coming months to years. The remote consequences of this could set up a background for neurodegeneration on which data are emerging in patients with Long‐COVID and can impose an enormous burden on an already weakened healthcare system because of the ongoing pandemic. Identification of biomarkers in CSF and serum that can alert when the damages are occurring at molecular levels in the CNS is important and urgently needed.^11^, ^12^

## CONFLICT OF INTEREST

The author has no conflict of interest to declare and no funding was obtained for this article.

## Data Availability

The data during the current study are available from the corresponding author on reasonable request.
